# Supplementary material for: Multiplexed phosphoproteomics of low cell numbers using SPARCE
Source: Commun Biol. 2025 Apr 26;8:666. doi: 10.1038/s42003-025-08068-x (PMC12033357; doi:10.1038/s42003-025-08068-x)
Supplement: Supplementary file 1 — Supplementary material [file 42003_2025_8068_MOESM1_ESM.pdf]

Multiplexed phosphoproteomics of low cell numbers using SPARCE

Emily J. Gaizley[1]*, Xiuyuan Chen[1]*, Amandeep Bhamra[1], Tariq Enver[1], Silvia Surinova[1#]

[1]UCL Cancer Institute, University College London, 72 Huntley Street, London WC1E 6BT, UK

*These authors contributed equally
[#]Correspondence: Silvia Surinova (s.surinova@ucl.ac.uk)

Supplementary Information

Contents

Supplementary Figure 1 p.2
Supplementary Figure 2 p.3
Supplementary Figure 3 p.4
Supplementary Figure 4 p.5
Supplementary Figure 5 p.6

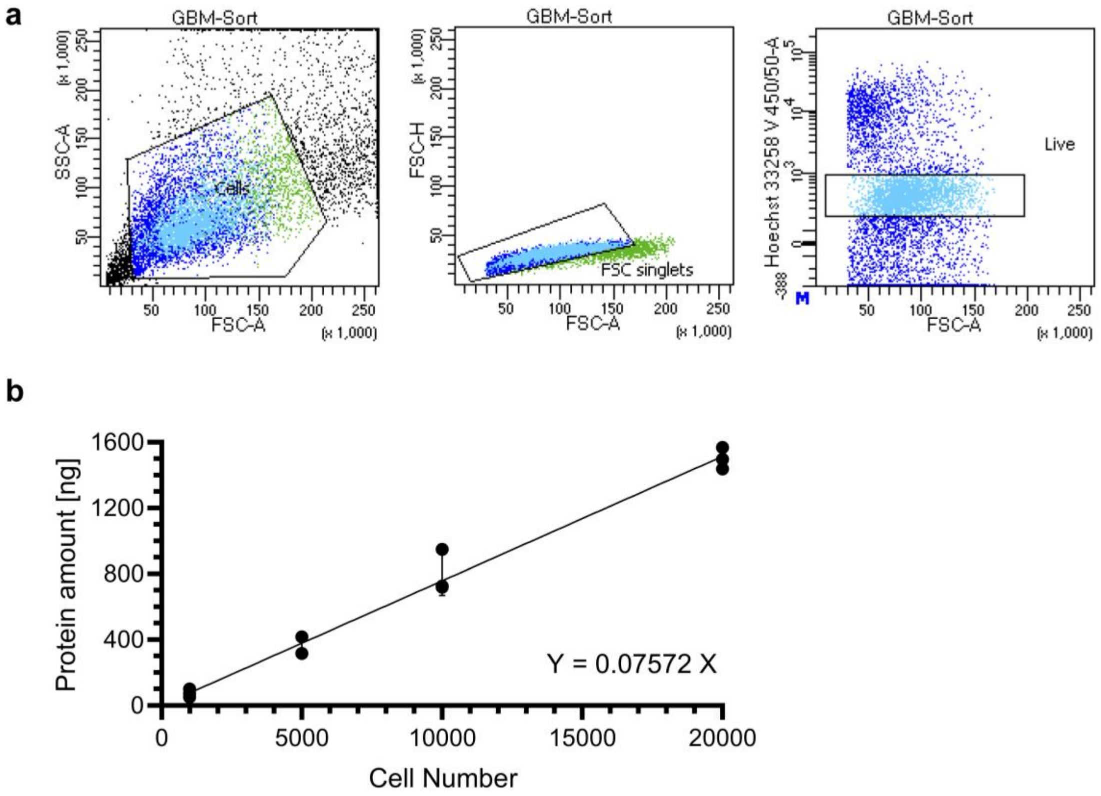

Supplementary Fig. 1 Quantification of protein yield from 1,000 GBM-E37 cells. a A representative FACS gating strategy to isolate live single cells. **b** Protein concentration curve for the calculation of protein amount in the GBM-E37 cell line. Data were calculated from a Micro BCA experiment using an independent standard curve and linear regression through (0, 0). A sample of 1,000 cells contains approximately 75 ng of protein (n=3).

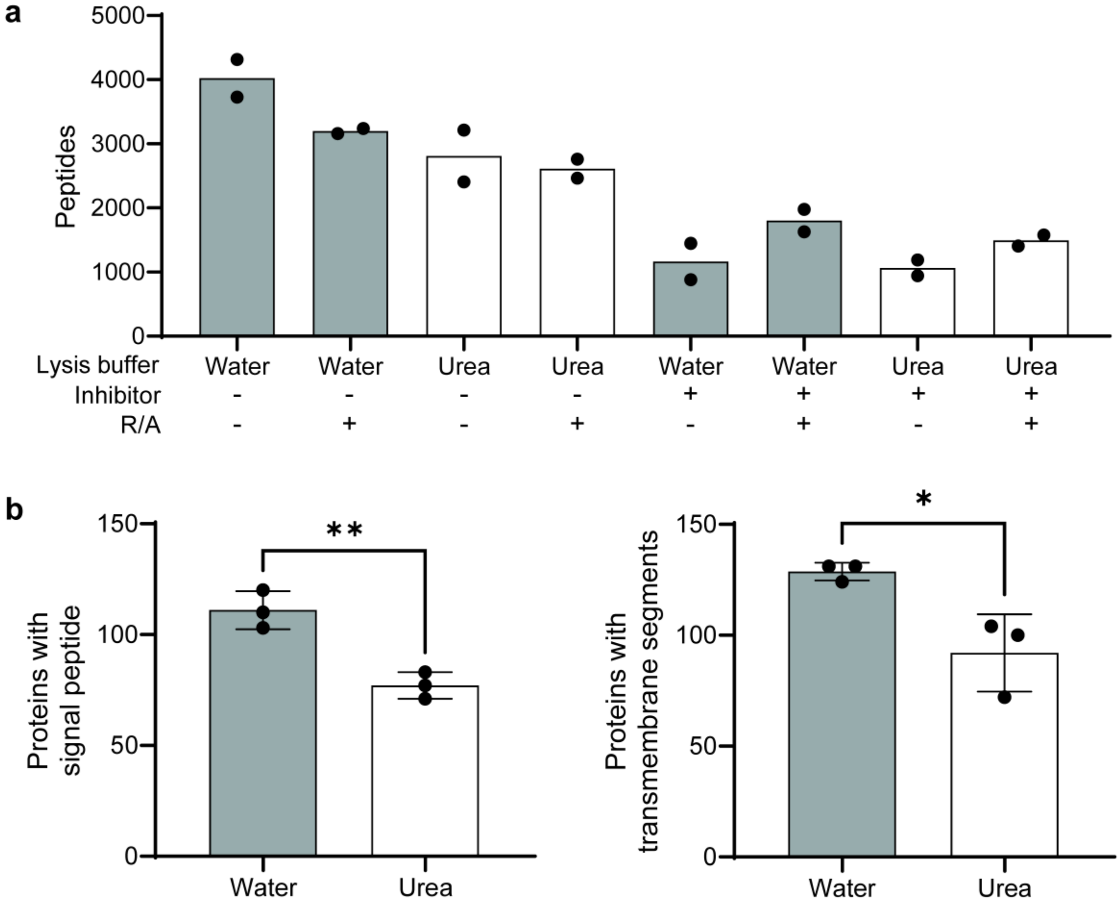

Supplementary Fig. 2 Optimisation of low-input sample lysis. a Bar plot comparing the total number of identified peptides under different lysis and digestion conditions: water-based or urea-based buffers, with or without protease inhibitors, and with or without reduction/alkylation (R/A). Each bar represents the mean of two replicates using unenriched samples (1,000 cells per sample) (n=2). **b** Bar plots illustrating the number of identified proteins with a signal peptide (left) and transmembrane segments (right), as annotated by Phobius. Results reflect comparisons between water-based and urea-based lysis in the absence of inhibitors and R/A. Each bar represents the mean of three replicates with error bars plotting the standard deviation (n=3).

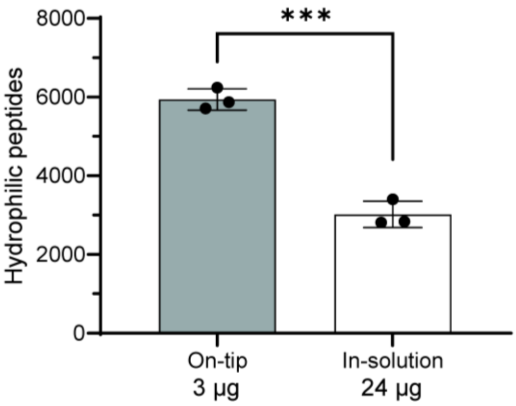

Supplementary Fig. 3 Comparison of on-tip and in-solution labelling methods for hydrophilic peptide identification. The total number of hydrophilic peptides identified from 1,000 GMB-E37 cells prepared with either on-tip labelling (3 µg TMTpro Zero label reagent) or in-solution labelling (24 µg TMTpro Zero label reagent). Hydrophilic peptides were defined using Grand Average of Hydropathy (GRAVY) scale. Each bar represents the mean of three replicates with error bars plotting the standard deviation (n=3).

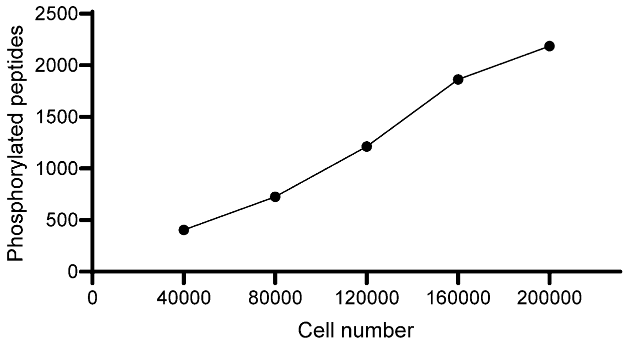

Supplementary Fig. 4 Optimising sample amount for multiplexed phosphopeptide enrichment. The relationship between different cell number inputs and identified phosphorylated peptides. Each data point represents a different cell input amount (ranging from 40,000 to 200,000 cells per sample) (n=1).

a

Plex name	Carrier size	Sample size	Carrier channel	"L12" cell line	"L21" cell line	"E37" cell line	"E53" cell line	Total protein input	Phospho-peptides
High input	N/A	20,000 cells	N/A	129N, 130N, 131N	129C, 130C, 131C	132C, 133C, 134C	133N, 134N, 135N	18 µg	3889
Low input	160,000 cells	1,000 cells	126C	129N, 130N, 131N	129C, 130C, 131C	132C, 133C, 134C	133N, 134N, 135N	13 µg	3044

b

	Low-input plex	High-input plex
**Minimum**	0.22%	0.18%
**Median**	21.84%	20.95%
**Maximum**	246.97%	413.21%

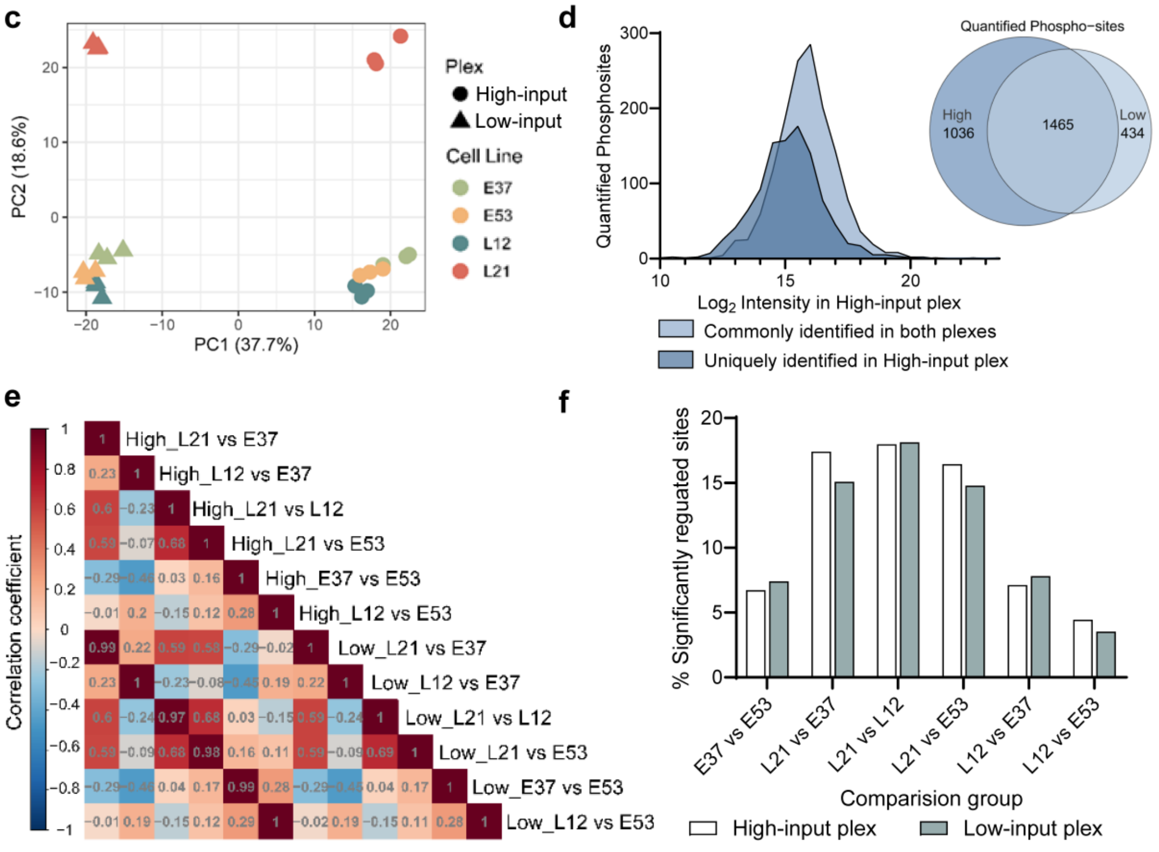

g

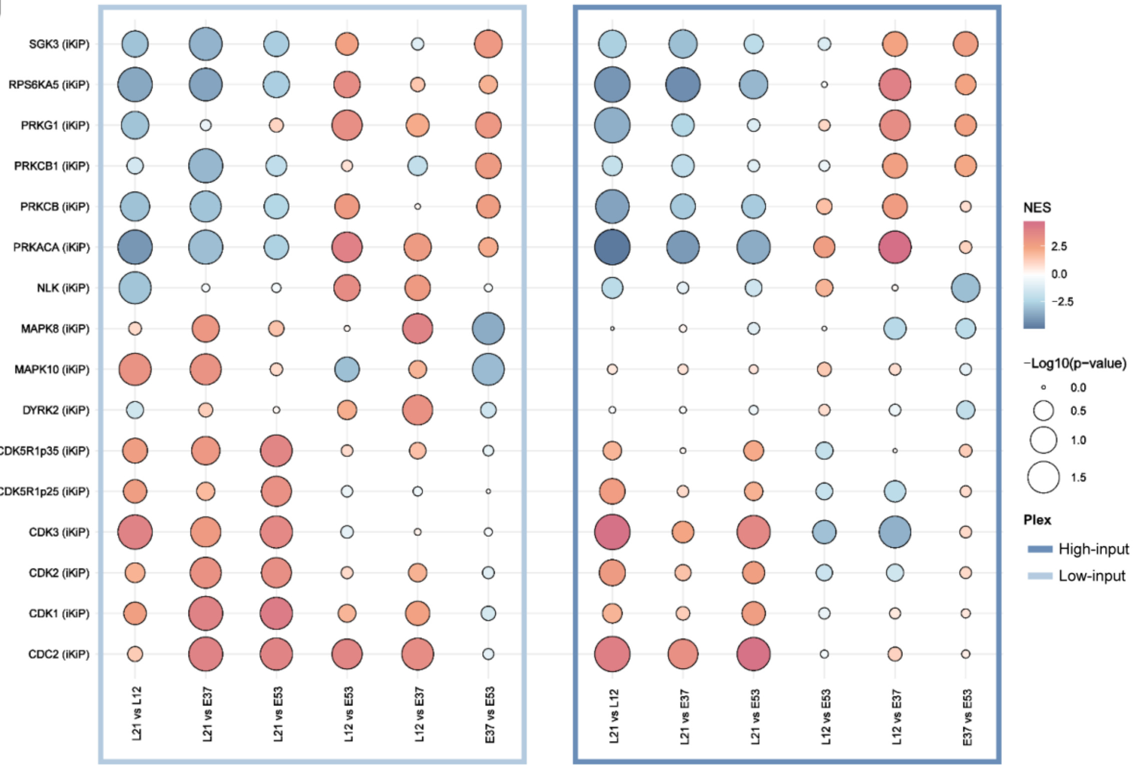

Supplementary Fig. 5 Comparison of the SPARCE workflow using different input amounts. a The experimental design to investigate the differences between four patient-derived glioblastoma cell lines. The table details the samples included in each plex, TMT labels used for each cell line, total protein input, and the number of phosphopeptides identified in each plex. **b** The maximum, minimum, and median CV values of the quantified phosphosites from both plexes. **c** Principal component analysis (PCA) of phosphosite abundances. PC1 (x-axis) and PC2 (y-axis) capture the major sources of variation in the dataset, highlighting how samples cluster according to cell line and plex. **d** Venn diagram (right) illustrating the number of phosphosites exclusively identified in the high-input plex (1,036), those identified exclusively in the low-input plex (434), and the overlap (1,465). The distribution plot (left) shows the abundance distribution of phosphosites commonly identified in both plexes (light blue) compared to those uniquely identified in the high-input plex (dark blue). **e** Correlation heatmap of pairwise comparisons across the four cell lines within each plex. The heatmap displays Pearson correlation coefficients of phosphosite fold changes, revealing the degree of similarity between different sample comparisons. **f** Phosphosite differential expression analysis comparing high-input and low-input plexes. Bars indicate the proportion of phosphosites that are significantly differentially expressed (adj. $p < 0.05$ and $|\log_2 FC| > 1$) across all pairwise comparisons of the four cell lines, highlighting the comparable fraction of regulated phosphosites identified at both input levels. **g** Inferred kinase activity profiles (bubble plots) for each comparison of the four cell lines. Kinase activity was ranked using PTM-SEA (Post-Translational Modification Set Enrichment Analysis). Kinases presented are from the iKiP database. Results from the low input plex are presented in the left panel, and the high plex is on the right. The bubble colour represents the normalised enrichment score (NES), ranging from negative (blue) to positive (red), while the bubble size corresponds to the $-\log_{10}$(p-value).
